# Data on endogenous chicken sperm peptides and small proteins obtained through Top-Down High Resolution Mass Spectrometry

**DOI:** 10.1016/j.dib.2016.07.050

**Published:** 2016-08-16

**Authors:** L. Soler, V. Labas, A. Thélie, A.P. Teixeira-Gomes, I. Grasseau, L. Bouguereau, E. Blesbois

**Affiliations:** a PRC, INRA, CNRS, Université de Tours, IFCE, 37380 Nouzilly, France; b ISP, INRA, Université de Tours, 37380 Nouzilly, France; c INRA, Plateforme d’Analyse Intégrative des Biomolécules, Laboratoire de Spectrométrie de Masse, 37380 Nouzilly, France

**Keywords:** Chicken, Sperm, Top-Down HRMS, Peptidome

## Abstract

The endogenous peptides and small proteins present in chicken sperm were identified in the context of the characterization of a fertility-diagnostic method based on the use of ICM-MS (Intact Cell Matrix-Assisted Laser Desorption/Ionization Time-of-Flight Mass Spectrometry). The interpretation and description of these data can be found in a research article, “Intact cell MALDI-TOF MS on sperm: a molecular test for male fertility diagnosis” (Soler et al., 2016) [1], and raw data derived from this analysis have been deposited to the ProteomeXchange Consortium via the PRIDE partner repository with the dataset identifier PRIDE: PXD002768. Here, we describe the inventory of all the molecular species identified, along with their biochemical features and functional analysis. This peptide/protein catalogue can be further employed as reference for other studies and reveal that the use of proteomics allows for a global evaluation of sperm cells functions.

**Specifications Table**

**Table:** 

Subject area	Male fertility diagnostics research

More specific subject area	Chicken (Gallus gallus) male gamete peptide/protein repository
Type of data	Figures, table
How data was acquired	Experiments performed on a LTQ orbitrap Velos Mass Spectrometer (Thermo Fisher Scientific, Bremen, Germany) coupled to an Ultimate®3000 RSLC Ultra High Pressure Liquid Chromatographer (Dionex, Amsterdam, The Netherlands)
Data format	Processed, analyzed
Experimental factors	Sample consisted of subfertile chicken sperm
Experimental features	Protein were extracted from chicken sperm and subjected to chromatographic fractionation prior to identification using Top-Down High Resolution Mass Spectrometry. Results were subjected to a functional analysis using bioinformatics tools.
Data source location	Nouzilly, Indre-et-Loire, France
Data accessibility	Data is within this article and accessible via the PRIDE partner repository at https://www.ebi.ac.uk/pride/archive/projects/PXD002768

**Value of the data**

• Here we make available for the scientific community the first repository of chicken sperm endogenous peptides and small proteins.
• This data describes Top-Down mass spectrometry protein identification results using two different pre-fractionation strategies: gel filtration and reverse phase chromatography, which can be useful when designing future identification strategies using the same technique.
• Data presented here include a description of the biochemical properties of the abovementioned identified sperm cells biomolecules as well as the molecular functions, biological processes and cellular components in which they are implicated.
• This information can be valuable in male fertility research studies.

## 1 Data

**Fig. 1:**
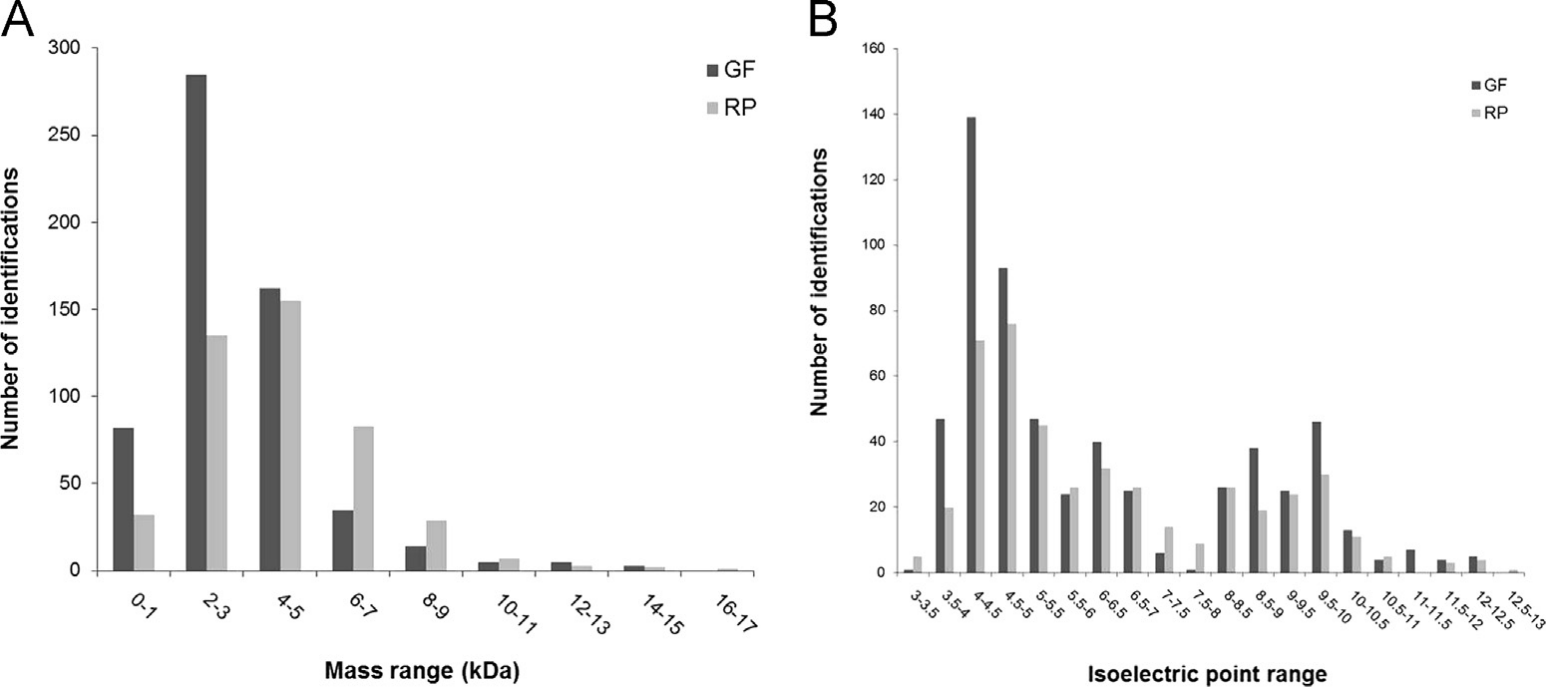
Comparison of the number of identifications achieved by Top-Down mass spectrometry in each mass (A) or isoelectric point (B) range for chicken sperm protein extracts separated either by gel filtration or reverse phase chromatography.

**Fig. 2:**
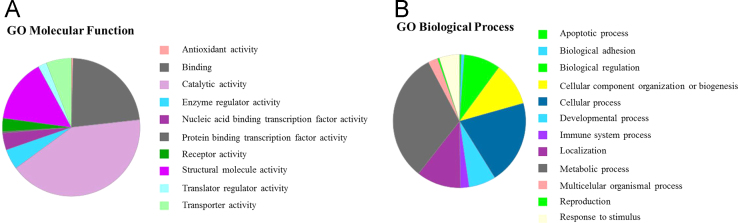
Molecular function (A) and biological process (B) of the biomolecules identified in chicken sperm by Top-Down mass spectrometry, according to the Gene Ontology classification.

This dataset consists of a compendium of peptidoforms and small proteoforms extracted from chicken ejaculated sperm, that were pre-fractionated using gel filtration or reverse phase chromatography and identified through a Top-Down mass spectrometry analysis. Hence, this set includes data regarding the identity of chicken sperm intact peptides and proteins as well as some structural biologically relevant information like N-terminal amino acids or post-translational modifications. See Fig. 1, Fig. 2 and Supplementary Table S1.

## 2 Experimental design, materials and methods

### 2.1 Experimental design and sample collection

This dataset was produced with the objective of identifying by Top-Down mass spectrometry the peptides and small proteins contained in chicken sperm cells [1]. Sperm cells (200 μL) protein extraction was performed by sonication in 400 µL of 6 M Urea 50 mM Tris–HCl pH 8.8 buffer containing protease inhibitor cocktail (Roche, Switzerland). Samples were centrifuged (45 min at 13,000 rpm and 4 °C) and supernatants containing the extracted proteins were kept for further analysis. The protein content was determined using a Bradford assay (BioRad, Marnes-la-Coquette, France).

### 2.2 Protein/peptide fractionation strategies, Top-Down mass spectrometry identification protocol and results

One mg of the extracted peptides/proteins were subjected to fractionation through chromatographic separation on an UltiMate 3000 RSLC system controlled by Chromeleon version 6.80 SR13 software (Thermo Scientific Dionex, Sunnyvale, USA) using reversed phase (RP) and gel filtration (GF) chromatography as described elsewhere [1]. A total of 35 and 65 fractions were obtained after RP and GF chromatography, respectively. All fractions obtained after fractionation were then analyzed by on-line micro-liquid chromatography tandem mass spectrometry (µLC-MS/MS) on a dual linear ion trap Fourier Transform Mass Spectrometer (FT-MS) LTQ Orbitrap Velos (Thermo Fisher Scientific, Darmstadt, Germany) coupled to an Ultimate® 3000 RSLC Ultra High Pressure Liquid Chromatographer (Thermo Scientific Dionex, Sunnyvale, USA) controlled by Chromeleon Software (version 6.8 SR11; Thermo Scientific Dionex, Sunnyvale, USA). Proteo/peptidoform identification and structural characterization were performed using ProSight PC software v 3.0 SP1 (Thermo Fisher Scientific, Darmstadt, Germany). The detailed procedure followed for this analysis is described elsewhere [1]. Raw data derived from Top-Down analysis have been deposited to the ProteomeXchange Consortium via the PRIDE partner repository with the dataset identifier PRIDE: PXD002768.

In total, 1038 intact or fragment protein masses were detected by Top-Down mass spectrometry (Supplementary data Table S1). A total of 447 biomolecules were detected in RP-derived fractions and 591 biomolecules in GF-derived fractions. From all the identified biomolecules, 65 were identified using both fractionation methods, while the rest was identified uniquely after RP or GF chromatography separation. The distribution of molecular weight and isoelectric point of the identified masses after each pre-fractionation method is represented in Fig. 1A and B, respectively.

## 3 Functional analysis

In order to identify which cell compartments/functions/pathways were mainly represented by the data set, a systems biology analysis was performed. In brief, the UniProtKB accession numbers from all *m/z* masses that were confidently identified through Top-Down analysis were recovered and listed. From these, the official human gene symbols (HuGO Gene Nomenclature Committee) were retrieved from *Gallus gallus* annotated proteins, whereas uncharacterized proteins were mapped to the corresponding *Homo sapiens* orthologs by identifying the reciprocal-best-BLAST hits. When manual annotation was needed (i.e. for deleted entries), this was performed by BLASTp (*E* value ≤e−04), together with Gene Ontology (GO) functional annotation. Because far more human genes are annotated and more information in databases is available for humans than for chicken, the human background was employed when possible. The functional classification tool from the Database for Annotation, Visualization and Integrated Discovery (DAVID version 6.7) website (https://david.ncifcrf.gov/home.jsp) was employed to group proteins contained in our dataset based on functional similarity (Supplementary Data Table S2). Functional analysis was also performed using the Panther Functional Classification System (http://pantherdb.org/) to evidence the most represented molecular functions (Fig. 2A) and biological processes (Fig. 2B). The functional analysis was completed using the “Set Distiller” module of GeneDecks (http://genecards.weizmann.ac.il/v3/index.php?path=GeneDecks; Supplementary Data Table S3).

The data presented here consist in a list of intact and unmodified (by chemical treatment) endogenous low molecular weight biomolecules (<15 kDa) present in chicken sperm. This dataset can be further employed as reference for other studies focused on the research of sperm cells.
